# Molecular markers from the chloroplast genome of rose provide a complementary tool for variety discrimination and profiling

**DOI:** 10.1038/s41598-020-68092-1

**Published:** 2020-07-22

**Authors:** Changhong Li, Yongqi Zheng, Ping Huang

**Affiliations:** State Key Laboratory of Tree Genetics and Breeding, Key Laboratory of Forest Silviculture and Tree Cultivation, State Forestry Administration, Research Institute of Forestry, Chinese Academy of Forestry, Beijing, 100091 China

**Keywords:** Genetics, Plant sciences

## Abstract

The rose is one of the most important ornamental woody plants because of its extensive use and high economic value. Herein, we sequenced a complete chloroplast genome of the miniature rose variety *Rosa* ‘Margo Koster’ and performed comparative analyses with sequences previously published for other species in the Rosaceae family. The chloroplast genome of *Rosa* ‘Margo Koster’, with a size of 157,395 bp, has a circular quadripartite structure typical of angiosperm chloroplast genomes and contains a total of 81 protein-coding genes, 30 tRNA genes and 4 rRNA genes. Conjunction regions in the chloroplast genome of *Rosa* ‘Margo Koster’ were verified and manually corrected by Sanger sequencing. Comparative genome analysis showed that the IR contraction and expansion events resulted in *rps19* and *ycf1* pseudogenes. The phylogenetic analysis within the *Rosa* genus showed that *Rosa* ‘Margo Koster’ is closer to *Rosa odorata* than to other *Rosa* species. Additionally, we identified and screened highly divergent sequences and cpSSRs and compared their power to discriminate rose varieties by Sanger sequencing and capillary electrophoresis. The results showed that 15 cpSSRs are polymorphic, but their discriminating power is only moderate among a set of rose varieties. However, more than 150 single nucleotide variations (SNVs) were discovered in the flanking region of cpSSRs, and the results indicated that these SNVs have a higher divergence and stronger power for profiling rose varieties. These findings suggest that nucleotide mutations in the chloroplast genome may be an effective and powerful tool for rose variety discrimination and DNA profiling. These molecular markers in the chloroplast genome sequence of *Rosa* spp. will facilitate population and phylogenetic studies and other related studies of this species.

## Introduction

Chloroplasts are vital and unique components of photosynthetic cells in plants and algae. They are responsible for multiple functions, e.g., assimilation of carbon and nitrogen, synthesis of amino acids and fatty acids, etc., and play important roles in plant growth and development^1^. Chloroplasts may have originated from cyanobacteria through endosymbiosis^2,3^, and chloroplasts possess their own deoxyribonucleic acid (DNA). In the majority of plant species, the chloroplast genome (cp genome) is inherited maternally, with the exception of certain gymnosperms, in which the cp genome is paternally inherited^4^. In recent years, knowledge about the organization and evolution of the cp genome has rapidly improved because of the development of DNA sequencing technology and bioinformatics methods. The cp genome is relatively conserved in angiosperms, and its size ranges from 107 to 218 kb, including 100–200 encoding genes^5^. A typical angiosperm cp genome has circular DNA, including two small inverted repeats (IRa and IRb), a large single copy (LSC) region and a small single copy (SSC) region^6^. Compared to nuclear DNA, the cp genome is small, single-stranded, relatively conserved and has uniparental inheritance, and its nucleotide mutation rate is moderate. Variation in the cp genome arises from the expansion or contraction of IRs and the length of the intergenic spacers^7,8^. The cp genome is considered to be conserved in the genome organization, gene order and gene content^9^; however, large-scale genome rearrangement and gene loss have been reported^10^. In addition, genes in chloroplast genomes can transfer to nuclear genomes and this process is part of the evolutionary process^11^. Hence, diverse DNA sequences from the cp genome have been used to study the evolution of plants^12^. In addition, the chloroplast sequences have contributed to assessing population genetic diversity, identifying species and implementing plant conservation^13–15^.

The rose is one of the most important woody ornamental plants and belongs to the genus *Rosa* in the family Rosaceae. *Rosa* species are native to temperate and warm regions in the Northern Hemisphere, especially in Asia. These species from different regions hybridize easily, giving rise to types that overlap the parental forms, and it is difficult to determine basic species^16^. Roses are widely used as cut flowers and in gardening and medicine, and they contribute to maintaining livelihoods, improving the environment and meeting material and spiritual needs. Therefore, roses have attracted much attention from plant breeders and botanists, and a large number of rose varieties have been selected for and bred^17,18^. Previous reports indicate that over 20 species in the *Rosa* genus have been used to breed modern rose varieties^19,20^. These intraspecific and interspecific hybridizations and artificial selections have not only created abundant variations but also led to complex genetic components in roses^21,22^. The increased number of varieties and decreased morphologic and genetic differences among varieties make it difficult to manage and discriminate rose varieties according to phenotypic differences, which is considered as the technical basis of distinctness, uniformity, and stability (DUS) examination in the current UPOV system. For example, rose plants must be submitted in the form of young plants that meet specific standards for field trials; these plants must be observed and examined for at least one growing cycle, based on 50 morphological characteristics^23^. However, the emergence of molecular markers may provide new insight into understanding and solving these practical problems because molecular markers can reveal addtional information on genetic variation. Previous reports have indicated that DNA evidence may provide exciting insight into the evolutionary process of *Rosa* species and identify the root of the original *Rosa* species and modern varieties^24–27^. Previous studies have reported that analyses of molecular markers from the nuclear genome are powerful and effective approaches for discriminating rose varieties and establishing DNA profiles. For example, random-amplified polymorphic DNA (RAPD), amplified fragment length polymorphism (AFLP) and simple sequence repeat (SSR)^28,29^, and sequenced tagged microsatellite site (STMS) markers have been used to construct the rose DNA database^30,31^. However, to date, only a few cp genomes of *Rosa* spp. have been published in the GeneBank database^32–35^, which is a source of data on sequence diversity. Furthermore, few studies have focused on molecular markers from the cp genome of roses. To identify hybrids, chloroplast markers alone may be insufficient, as these markers are haploid and only provide maternal data; nevertheless, these markers may be useful tools for identifying a maternal parent of putative hybrid progeny. Using the associated cp genome sequence in combination with nuclear genomic markers to construct rose DNA profiles could be helpful in the management and discrimination of varieties.

In this paper, we hypothesized that the molecular markers and sequence diversity of the cp genome may assist in discriminating and profiling modern rose varieties. Therefore, we constructed a complete cp genome of *Rosa* ‘Margo Koster’ using next-generation sequencing with a de novo and reference-guided assembly strategy. The cp genome of *Rosa* ‘Margo Koster’ was compared and analyzed with the genomes of other species or varieties in the *Rosa* genus. Moreover, a complete cp genome of *Rosa* ‘Margo Koster’ and characterizations of SSRs and single nucleotide variations (SNVs) were screened and verified by capillary electrophoresis (CE) and Sanger sequencing, and their discriminating power was calculated and compared in a set of rose varieties.

## Results

### Genome sequence generation and PCR-based validation

More than 1,190.28 million reads (approximately 11 Gb of clean data) were generated by the Illumina HiSeq2500 platform for *Rosa* ‘Margo Koster’. These data were used to assemble the cp genome with a high mean coverage. After de novo and reference-guided assembly, the complete cp genome with a size of 157,395 bps was generated. Four regions and their conjunctions were validated using PCR-based Sanger sequencing, and we also corrected the errors via PCR-based validation. We designed 35 pairs of primers based on the variation in regions of alignments to validate these sequences (see Table S1); the gel photo of the PCR products are shown in Figure S1. The validated sequences amounted to 28,662 bp. We compared these sequences to the assembled genome and found two nucleotide mismatches that were corrected before submission to the GenBank database (accession No. MN435990).

### General cp genome characterization and annotation

**Figure 1:**
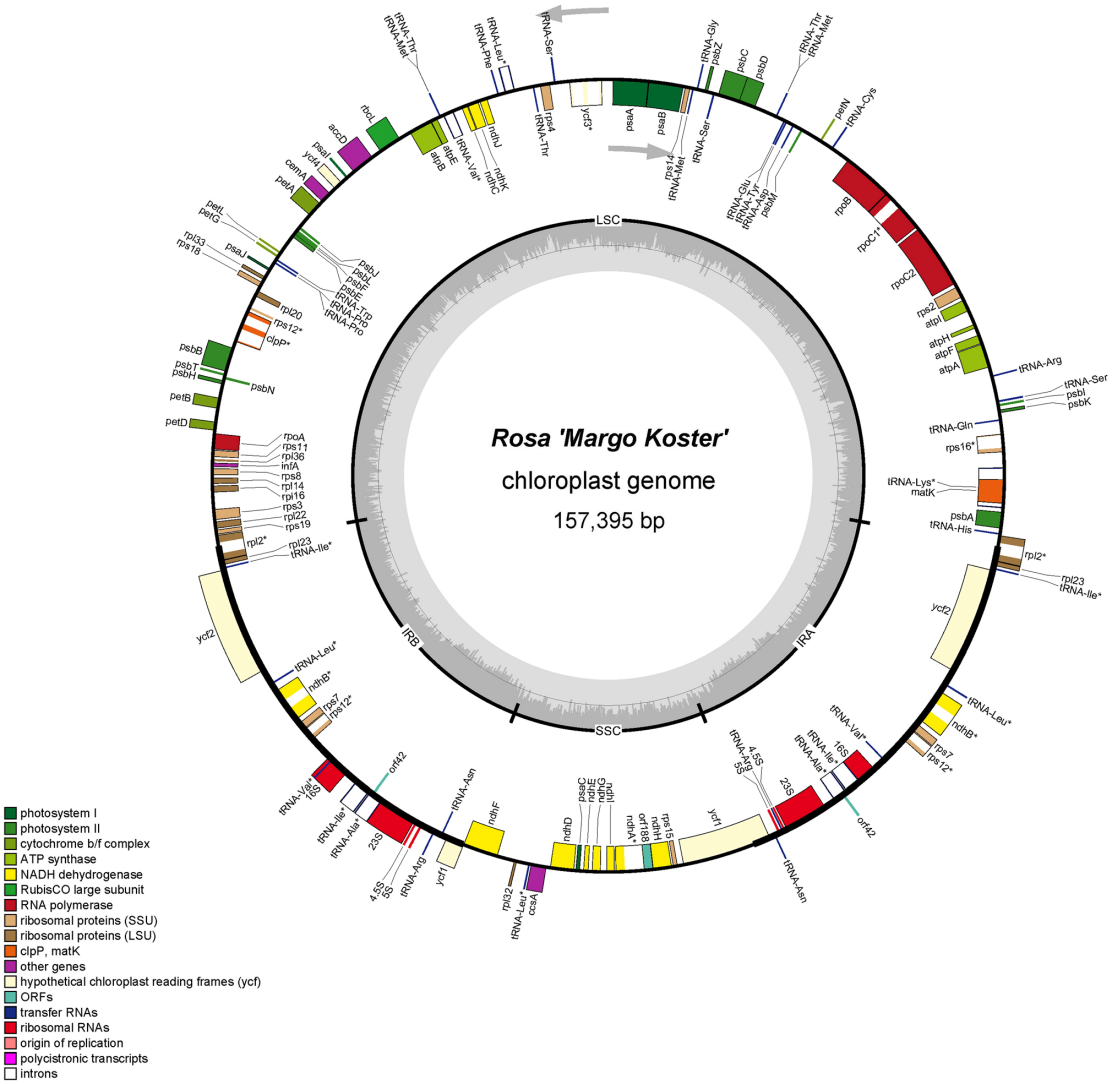
Circular map of the cp genome for *Rosa* ‘Margo Koster’. The gray arrows show that genes inside the circle are transcribed clockwise, and genes outside the circle are transcribed counterclockwise. The innermost shaded areas inside the inner circle correspond to the GC content in the cp genome. Genes in different functional groups are color coded. The boundaries of four regions (IRa, IRb, LSC, SSC) are noted in the inner circle.

The cp genome displayed a typical quadripartite structure, consisting of one LSC region with a length of 87,710 bp, one SSC region with a length of 18,849 bp and a pair of IR regions (IRa and IRb) with a length of 25,418 bp each (Fig. 1). The overall GC content of the cp genome was 37.19%. Among the LSC, SSC and IR regions, the highest GC content was found in the IR regions (43.34%), and the GC content of the LSC and SSC regions was 35.19%, and 31.20%, respectively. More rRNA and tRNA genes, which have high GC content, could explain the higher GC content in the IR region than other regions. Thymine (T) and adenine (A) preferences in the third position of the codon were observed in this cp genome, and codon usage is shown in Table S2 and Figures S2 and S3. This event could be a result of an A + T rich genome, which has been also observed in other plants^36–38^.

The gene content and order are illustrated in Fig. 1 and Table 1. There are a total of 115 genes, including 81 protein-coding genes, 30 tRNA genes and 4 rRNA genes. Seven protein-coding genes, 7 tRNA genes, and 4 rRNAs were duplicated in the IR regions. The LSC region contained 22 tRNA genes and 63 protein-coding genes, and the SSC region included 11 protein-coding genes and 1 tRNA gene. Most of these protein-coding genes did not contain introns, except that nine genes *(rps16, rpoc1, trnI-GAU, trnA-UGC, rps12, rpl16, ndhA, rpl2, ndhB)* contained a single intron and two genes (*ycf3*, *clpP*) contained two introns. The longest intron was *trnK-UUU,* with a length of 2,499 bp, and was present in the *matK* gene.

**Table 1:** The gene content and functional classification in the cp genome of *Rosa* ‘Margo Koster’.

Function	Genes
RNA transfer	trnH-GUG, trnK-UUU, trnQ-UUG, tRNA-Gly, trnG-UCC, trnR-UCU,trnD-GUC, trnY-GUA, trnE-UUC, trnT-GGU, trnM-CAU, trnS-UGA, trnfM-CAU, trnS-GCU, trnS-GGA, trnT-UGU, trnL-UAA,trnF-GAA,trnV-UAC, trnW-CCA, trnP-UGG, trnP-GGG, trnI-CAU, trnL-CAA, trnV-GAC, trnI-GAU*, trnA-UGC*, trnN-GUU, trnR-ACG, trnL-UAG
RNA ribosomal	rrn23, rrn16, rrn5, rrn4.5
RNA polymerase	rpoC1*, rpoC2, rpoA, rpoB
Clp^p^, Matk, ORFs	Clp^p^**, matk, orf42, orf188
Ribosomal proteins (SSU)	rps2, rps3, rps4, rps7, rps8, rps11, rps12*, rps14, rps15, rps16*, rps18, rps19
Ribosomal proteins (LSU)	rpl2*, rpl2^Ψ^, rpl14, rpl16, rpl20, rpl22, rpl23, rpl32, rpl33, rpl36
Hypothetical chloroplast reading frames (ycf)	ycf1, ycf1^Ψ^, ycf2, ycf3**, ycf4
ATP synthase	atpE, atpB, atpA, atpF, atpH, atpI
Photosystem I	psaI, psaB, psaA, psaC, psaJ
Photosystem II	psbD, psbC, psbZ, psbT, psbH, psbK, psbI, psbJ, psbF, psbE, psbM, psbN, psbL, psbA, psbB
RubisCO large subunit	rbcL
Cytochrome complex	petN, petA, petL, petG, petB, petD
NADH dehydrogenase	ndhB*, ndhI, ndhK, ndhC, ndhF, ndhD, ndhG, ndhE, ndhA*, ndhH, ndhJ
Others	infA, accD, cemA, ccsA

*Genes containing one intron; **genes containing two introns; ^Ψ^pseudogene.

### Phylogenetic analysis in Rosaceae

**Figure 2:**
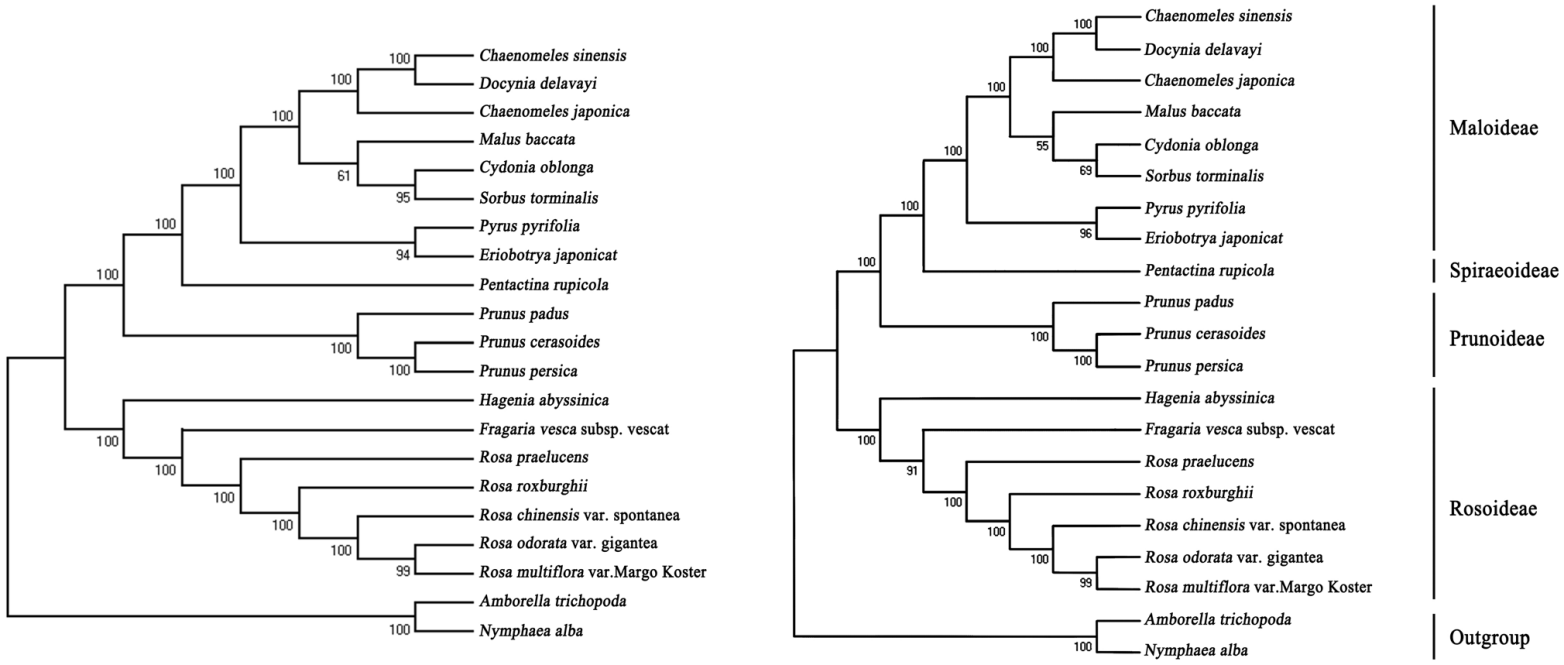
Neighbor-joining (NJ) and maximum likelihood (ML) trees for the Rosaceae family inferred from the complete cp genomes of 19 species from 5 subfamilies. Bootstrap values are indicated at the nodes. The length scale behind the tree indicates substitutions per site.

Sequencing alignment showed that the cp genomes of *Rosa* species are more homologous than those of other species of Rosaceae. Phylogenetic trees illustrate the partial relationship of some species in Rosaceae (Fig. 2) based on the maximum likelihood (ML) and neighbor-joining (NJ) methods. The results were consistent with previous studies of Rosaceae species^39^, and showed that *Rosa* ‘Margo Koster’ has a closer genetic similarity to *Rosa odorata* var. gigantea and *Rosa chinensis* var. spontanea than with the other *Rosa* species, which may be attributed to the selection of breeding material.

### Comparison with the cp genomes from *Rosa* species

**Figure 3:**
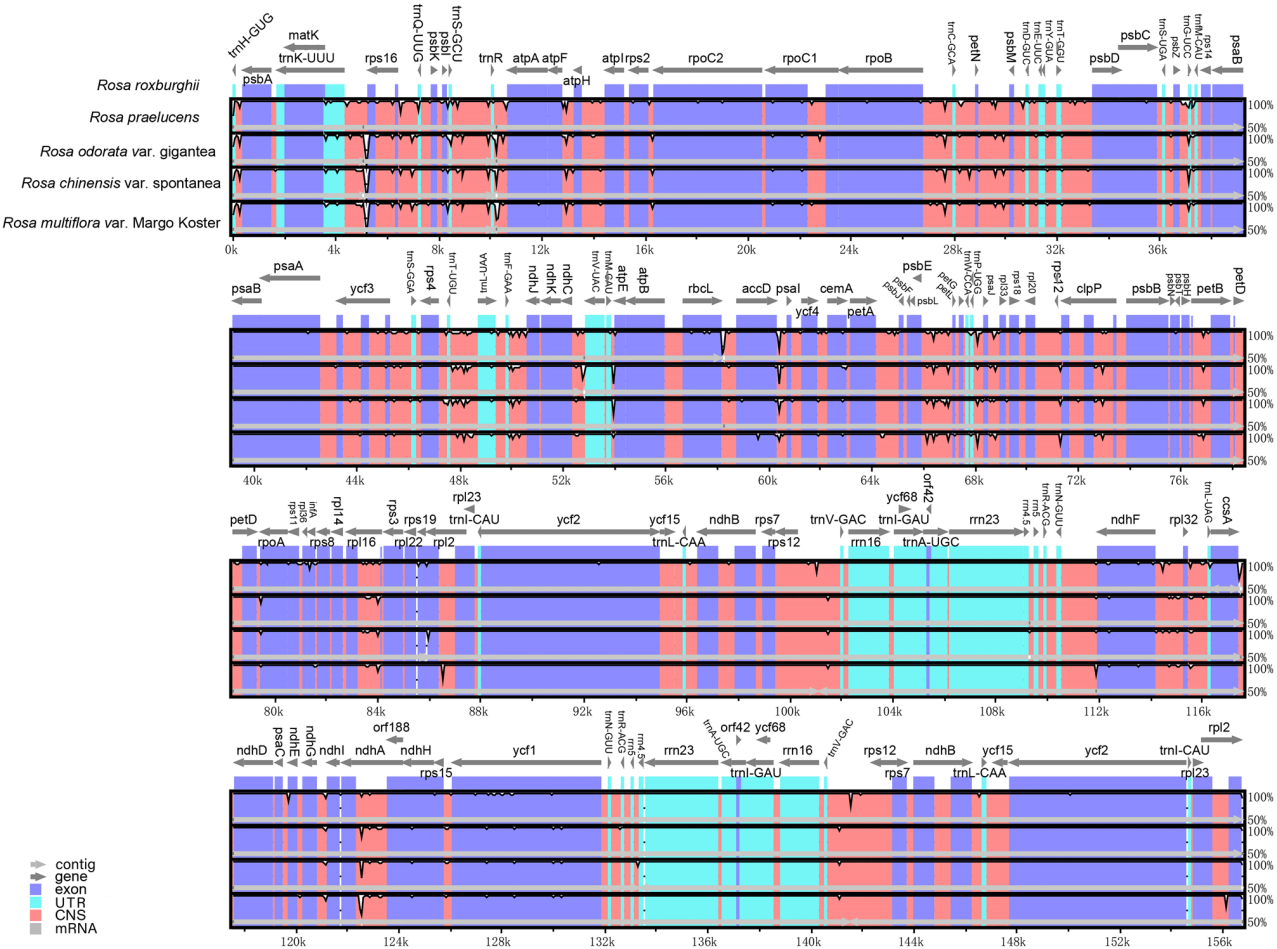
Comparisons of sequence identity of cp genomes for five *Rosa* species. The vertical axis represents identity ranging from 50 to 100%. Each arrow indicates the annotated gene and its transcriptional direction. Genome regions are color coded as an exon, mRNA or tRNA, untranslated region (UTR) and conserved noncoding sequence (CNS).

The whole cp genome of *Rosa* ‘Margo Koster’ was compared with those of four *Rosa* species, and the highly divergent regions are shown in Fig. 3. As a whole, the shared sequence identities were over 95% in pairwise comparisons for the five *Rosa* species. The sequences in two IR regions were more conserved than those in LSC and SSC regions, and protein-coding regions were less divergent than noncoding regions, such as introns and intergenic regions. The highly divergent regions were mainly found in intergenic regions, including *trnD-GUC-trnY-GUA, rpoB-trnC-GCA, rpl12-clpp, aptF-aptH, rps2-rpoc2, trnS-GGA-rps4, psaJ-rpl33, rps16-trnQ-UUG, trnK-UUU-rps16, psbE-petL, trnT-UGU-trntL-UAA, trnP-UGG-psaJ, trnH-GUG-psbA, trnR-UCU-atpA, psbZ-trnG-UCC, trnG-UCC-trnfM-CAU, trnM-CAU-aptE, psbM-trnD-GUC,* and *psbL-trnS-GCU*. Apart from those regions, some coding sequences, such as *ndhA, clpp, ndhF, petB,* and *ycf1*, had low similarity levels.

**Figure 4:**
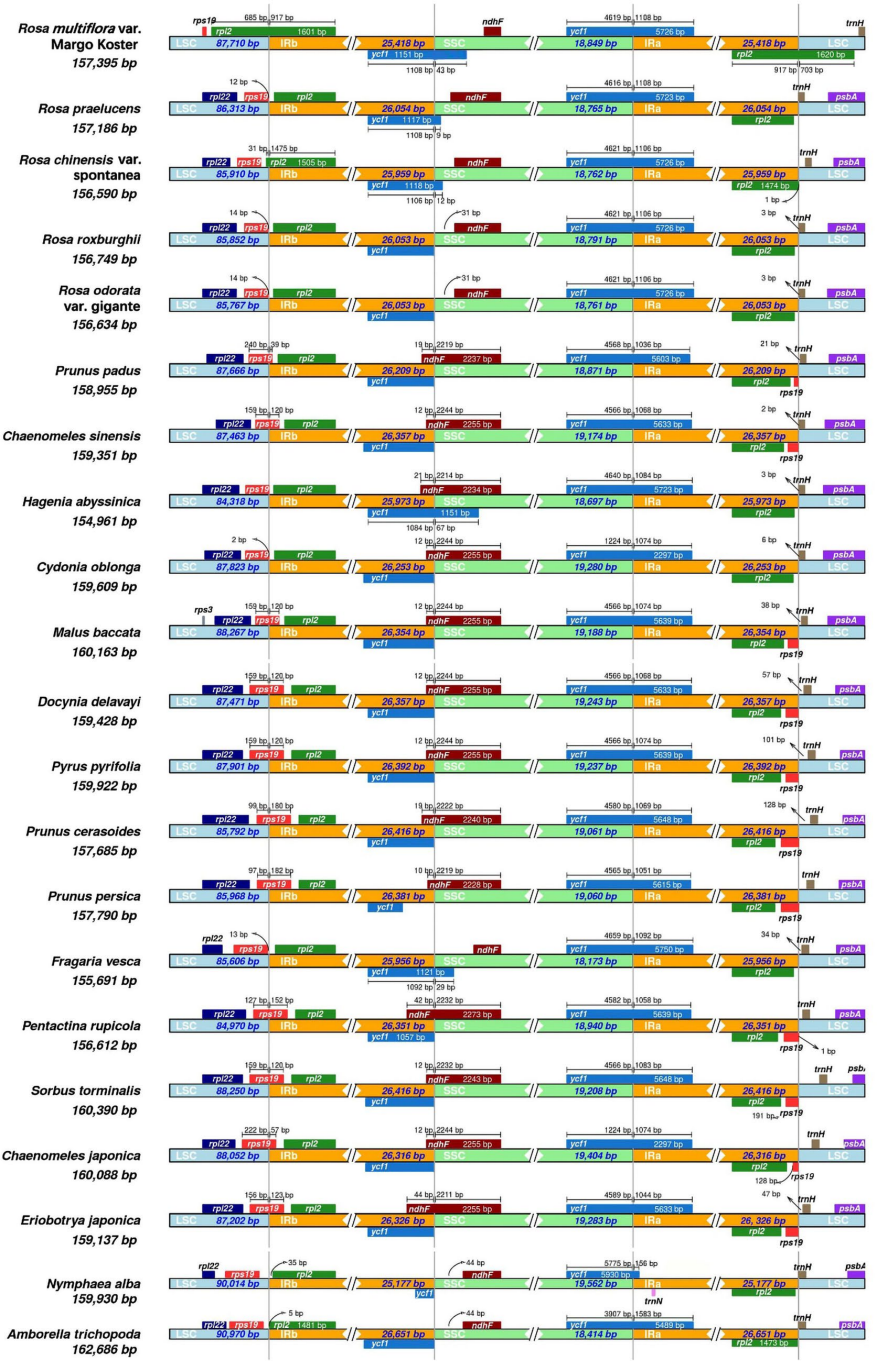
Comparison of the boundaries between LSC, SSC, and two IR regions among 19 cp genomes within the Rosaceae family and two species of basal angiosperms. The boundaries of five *Rosa* species are shown together at the top of the figure, and the differences in their boundaries are obvious. The number of base pairs (bp) represents the distance from the boundary to the end of the gene.

A comparison among boundary regions of cp genomes is illustrated in Fig. 4, and the list of 19 species in the family of Rosaceae and 2 basal angiosperm species is shown in Table S3. The results showed that the position of the SSC/IRb junction in all species was the *ycf1* gene, and a pseudogene near the 5′ end of this gene (*yfc1*^Ψ^) was found in the IRa region. The size of *yfc1*^Ψ^ was approximately 1,100 bp for the Rosaceae species. The *rpl2* gene stretches across the boundary between the LSC and IRb regions and was only found in *Rosa chinensis* var. spontanea and *Rosa* ‘Margo Koster’, not in the other three species, which all contained a complete *rpl2* gene in the IRb region. The *rps19* gene was located in the LSC region in the five *Rosa* species and in one *Fragaria* species, but a partial sequence of this gene entered the IRb region in *Malus*, *Pyrus*, *Prunus* and other species. Therefore, a pseudogene (*rps19*^Ψ^) was designated in the IRa region. The *ndhF* gene was located completely within the SSC region for five *Rosa* species and the *Fragaria* species; however, the *ndhF* gene was located at the boundary of the LSC and IR regions in the other Rosaceae species, e.g., *Pentactina rupicola*, *Chaenomeles japonica*, and *Sorbus torminalis.*

### Repeat sequence analysis of *Rosa* species

A total of 84 repeat sequences were detected in the cp genome of *Rosa* ‘Margo Koster’, containing 27 tandem repeats, 26 forward repeats, one complementary repeat, 4 reverse repeats and 26 palindrome repeats. The detailed information is shown in Tables S3 and S4. SSRs, which are composed of a 1–6 bp low-repeat motif, were also widely found in the genome. A total of 49 perfect SSRs were detected, including 46 mononucleotides and 3 dinucleotides. Over 89% of the SSRs were mononucleotides, which contained a repeat motif of adenine (A) or thymine (T). For dinucleotide SSRs, the repeat motif was a repetition of only AT. The results showed that 85.71% of all the detected SSRs were located in the LSC regions, 6.12% in the IR regions, and 8.16% in the SSC regions. These findings indicated that the distribution of cpSSRs was not imbalanced in the cp genome of *Rosa* ‘Margo Koster’.

### Highly divergent regions among *Rosa* species

The level of divergence among five *Rosa* species was variable in different regions of the cp genome. The results showed that the nucleotide diversity (π) ranged from 0.0012 (*rps12-trnV-GAC*, intergenic space) to 0.0524 (*psbL-trnS-GCU*, intergenic space) based on the comparative analysis of the regional sequence. The results showed that the average π value of the intergenic space was 0.0108, which was higher than that of the protein-coding region (0.0053), and genetic divergence did not exist in the rRNA sequences (π = 0). These findings indicated that the level of genetic variance depended on the region. Twenty regions with the highest π values are shown in Table 2, and their sequences included 19 IGSs and only one protein-coding gene—the *ndhA* gene—with a π value of 0.0094. Other regions with π values greater than zero are also illustrated in Figure S4.

**Table 2:** Twenty most divergent regions of the cp genome based on a comparison of *Rosa* species.

No.	Region	Nucleotide diversity (π)	Total number of mutations (η)	Region length (bp)
1	*ndhA*	0.0094	41	2,381
2	*trnD-GUC-trnY-GUA*	0.0100	10	420
3	*rpoB-trnC-GCA*	0.0101	27	1,220
4	*rpl12-clpp*	0.0101	4	201
5	*aptF-aptH*	0.0103	12	510
6	*rps2-rpoc2*	0.0109	5	247
7	*trnS-GGA-rps4*	0.0110	7	292
8	*psaJ-rpl33*	0.0113	13	469
9	*rps16-trnQ-UUG*	0.0120	21	883
10	*trnK-UUU-rps16*	0.0121	20	827
11	*psbE-petL*	0.0124	35	1,314
12	*trnT-UGU-trntL-UAA*	0.0126	32	1,240
13	*trnP-UGG-psaJ*	0.0192	18	450
14	*trnH-GUG-psbA*	0.0244	11	312
15	*trnR-UCU-atpA*	0.0244	21	631
16	*psbZ-trnG-UCC*	0.0320	23	400
17	*trnG-UCC-trnfM-CAU*	0.0323	12	223
18	*trnM-CAU-aptE*	0.0368	9	214
19	*psbM-trnD-GUC*	0.0492	62	529
20	*psbL-trnS-GCU*	0.0524	18	183

### Development and utilization of molecular markers in chloroplast genomes of *Rosa* species

**Figure 5:**
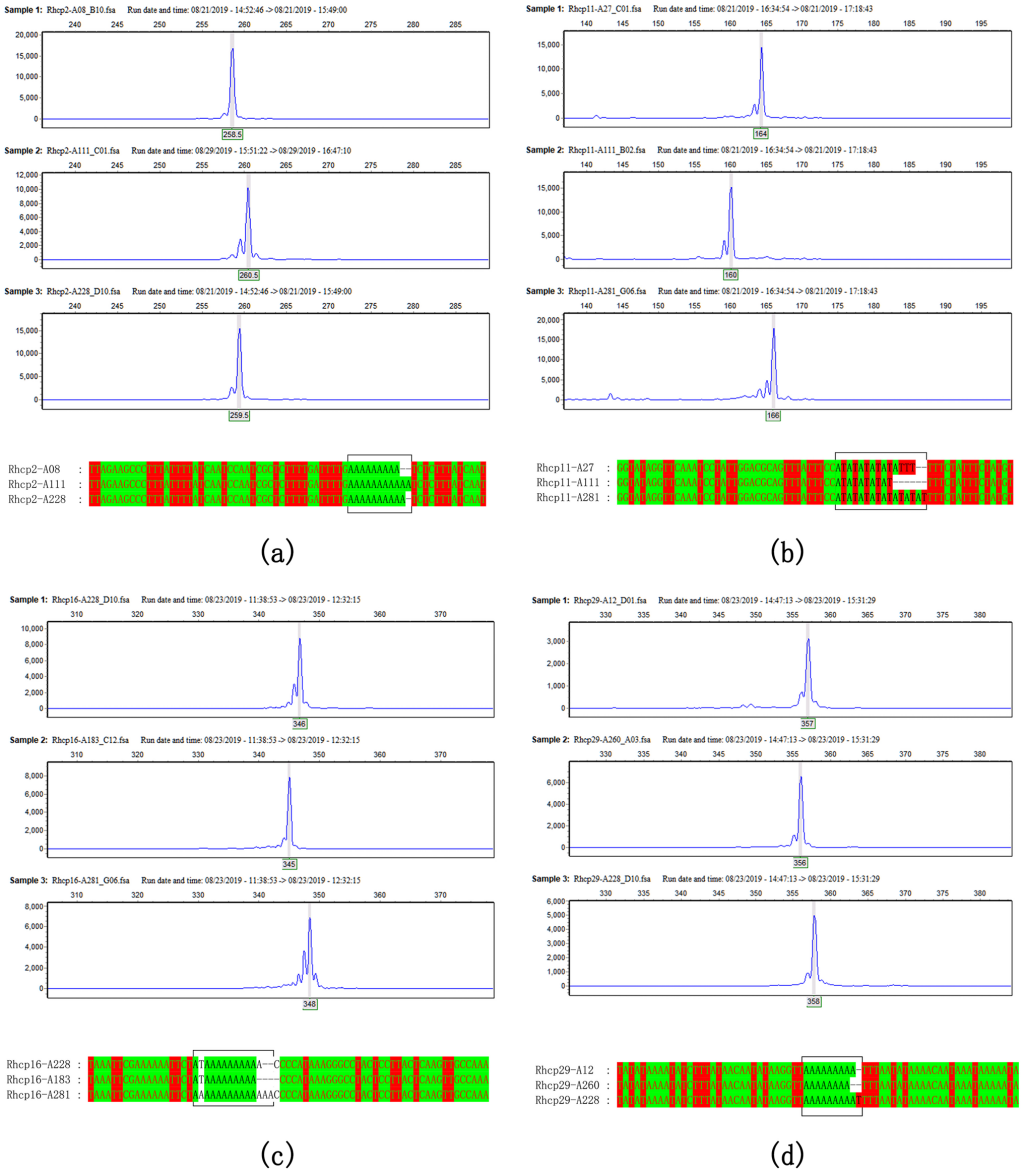
The cpSSR genotype of rose varieties based on capillary electrophoresis and Sanger sequencing. (**a**)–(**d**) Variation in fragment size of cpSSRs and their repeat motif in different rose varieties.

Based on repeat sequence analyses, a total of 49 candidate cpSSRs were subjected to genotyping using CE and Sanger sequencing in a set of different original rose varieties. The results showed that clear, stable and expected PCR products were obtained in all cpSSR loci; however, only 15 cpSSR loci were abundantly polymorphic, accounting for 31.64% (Table 3, Fig. 5). The results showed that the D_j,_ value, which is the discriminating power, ranged from 0.1043 to 0.9350, with an average value of 0.4018, based on the genotyping of cpSSRs (Table 3). These findings indicated that cpSSRs could be moderately polymorphic and have discriminating power in the set of test rose varieties. Over one hundred SNVs were also found in the flanking sequence of cpSSRs. The results showed that the π among the test rose varieties ranged from 0.0089 to 0.0744, with an average value of 0.0315, and the total number of mutations (**η**) ranged from 3 to 150. The D_j_ values ranged from 0.3446 to 0.9996, with an average value of 0.8303. These findings indicated that these SNVs in the cp genome have much more polymorphism and stronger discriminating power than traditional fragment-size molecular markers. Additionally, all the polymorphic cpSSRs and SNVs were used to examine and construct DNA profiles of 93 rose varieties.

**Table 3:** The Primer information of 15 cpSSRs and nucleotide mutations of their flanking sequences.

Locus Name	Forward primer	Reverse primer	Product size (bps)	Tm (°C)	Nucleotide diversity (π)	Total number of mutations (η)	Region length (bp)	D_j_^a^	D_j_^b^	Site
Rhcp2	AACTCATCAACGGACTCTCCA	ATTAGTGCTTGATGCGGGAAA	261	59.5	0.00193	3	205	0.4130	0.3446	*matK-trnK-UUU*
Rhcp5	GACTTGTGTTGGATTGGCACT	ACGGAACTTCGCCTTAACCAA	289	60	0.02362	53	221	0.1231	0.9016	*trnK-UUU-rps16*
Rhcp6	CATTCCTTCAGTTTGGAACCCA	TCTTGGTACTTGAAGAAGTGTGA	337	58	0.06351	150	271	0.4544	0.9885	*rps16-trnQ-UUG*
Rhcp11	TGGAGTGAAAAGCGTCCATTG	AGCGCCTCTTATTCAAGTTATTCA	167	59	0.04529	78	113	0.3111	0.5339	*trnR-UCU-aptA*
Rhcp13	CGGATGGCCAATAACCCAAG	GAGGTATTTCGCAACTGGCCT	311	59	0.00906	58	266	0.3700	0.5196	*atpF-atpH*
Rhcp14	CGCACGTCGTAAACAAATCCA	AGCTTAGCCTGACGCAATGT	331	60	0.05264	76	282	0.4642	0.8277	*atpH-aptL*
Rhcp16	CCGGCTCCAGTAGTTACACC	TAACCGTCGAGGCGAAGTAG	350	60	0.00891	34	296	0.2170	0.9495	*rpoC1*
Rhcp19	CACATATTGCGCACTTCCCG	AGGGCCTCTTCGATGGGTAT	246	60	0.04801	48	200	0.3534	0.9996	*petN-psbM*
Rhcp22	CGCTATCCGCCCAGGATAAT	CCTTGAGGTCACGGGTTCAA	243	60	0.01792	24	197	0.3020	0.9727	*trnG-UUC-trnfM-CAU*
Rhcp24	CGGGGATACACGACAGAAGG	CACCTATTACAGAGATGGTGCGA	190	60	0.02778	23	114	0.1043	0.9084	*ycf3*
Rhcp29	CGTGTAGAAACGTGTAGAAGGG	GAAACCATTGCAATTGCCGGA	360	59	0.01671	48	322	0.4523	0.7457	*accD-psaL*
Rhcp31	AGCGAGTCAACCGCTAGAAC	GGAGAATGAACTCTGGGAAGGT	296	60	0.03396	53	252	0.5046	0.9991	*rps18-rpl20*
Rhcp34	CTCCGAGTAAAGATCCGCCC	TGAAGTATCCAGGCTCCGTT	308	59	0.03085	92	272	0.3925	0.7929	*rpl 20*
Rhcp38	TGTGTATCTAGGGAATCGTCGC	CTGCCCCCGAGGGTCTATAA	319	59	0.07443	81	269	0.9350	0.9930	*petD-rpoA*
Rhcp41	AGCTCCTCGCGAATTAAACGA	TGGGAACGACAGAACCTGTG	390	60	0.01791	39	352	0.6307	0.9790	*rpl 16-rps3*

‘a’ indicates the discriminating power based on the genotype of cpSSR; ‘b’ indicates the discriminating power based on the genotype of nucleotide mutations.

## Discussion

In this paper, we constructed cp genomes for a rose variety (*Rosa* ‘Margo Koster’) using next-generation sequencing on the Illumina HiSeq2500 platform. The comparative analysis of this cp genome and those of other species in the Rosaceae family provided new insight into the characteristics of their cp genomes, and the identified polymorphic markers in these cp genomes could be suitable for use in future DNA profiling, variety discrimination and evolutionary studies in *Rosa* spp. and their varieties to manage and profile a large number of rose varieties, clarify genetic relationships, and assist in breeding selection.

Compared to previously published data in Rosaceae, the cp genome of *Rosa* ‘Margo Koster’ has a typical quadripartite structure, where the LSC and SSC regions are separated by two IRs and show a relative similarity in cp genome size, gene number and structure, as well as total GC content, which is an extremely significant indicator of species affinity^40^. Compared with genomes of other genera, no large fragment inversions or gene rearrangements were detected in the *Rosa* genus. Noncoding sequences in the IR regions were more conserved than others that have been found in the cp genomes of Asteraceae and Lamiales^41,42^. Therefore, more genetic variation has originated from the LSC and SSC regions than from IR regions.

The contraction and expansion of IR regions, as an important evolutionary event in the cp genome development, is one of the primary reasons for the variation in size of the cp genome^43,44^. Therefore, the length of the IR region is variable, and this region is even lost in some plant species, such as *Pelargonium* × *hortrum*, *Pinus thunbergii*, *Cryptomeria japonica*, *Erodium* and *Metasequoia glyptostroboides*^45–49^. This type of gene arrangement in the cp genome is different from that of most eudicots and monocots; for example, in eudicots, the *trnH* gene is located in the LSC region rather than the IR region, where this gene is located in most monocots^50^.

Another important aspect of the use of the cp genome in evolutionary studies is the variation found near the boundaries among the four regions. The IR contraction and expansion could provide evidence about the evolution of some plant taxa and lead to the size or sequence divergences of the cp genome. Our comparative analysis of 19 species in the Rosaceae family showed that the *rpl2* gene in *Rosa* ‘Margo Koster’ has moved from an IR region to an LSC region, resulting in a shorter IR region than that of other *Rosa* species, and this phenomenon of IR contraction has also occurred in *Rosa chinensi*s var. Spontanea but not in other three *Rosa* species. Our results also revealed that some pseudogenes contribute to the variation in length in the IR region, such as in *rps19*^Ψ^ or *ycf1*^Ψ^, and their biological function is still unclear. For example, *ycf1,* which is one of the largest ORFs in the cp genome, has been considered a protein-coding gene in tobacco, and the protein encoded by the *ycf1* gene has been found to be important for cell survival or related to the ABC transporter in other studies^51,52^; *ycf1*^*Ψ*^ is present in all studied species. The *rps19* gene is present in the cp genome of Rosaceae species, and its location varies from species to species; for example, the *rps19* gene is located in the LSC region in *Rosa* and *Fragaria* species, whereas this gene stretches across the LSC and IR regions and may lead to incomplete duplication in *Malus*, *Prunus* and *Pyrus* species, resulting in *rps19*^*Ψ*^. Contraction and expansion of the IR region have taken place in Rosaceae, leading to the creation of pseudogenes.

Repetitive sequences play an important role in genome recombination and rearrangement; thus, they are major sources of genetic variation. Many repetitive sequences have been identified and applied in a wide range of studies in plant science^53–56^. However, little attention has been given to cpSSRs in the cp genome of *Rosa* species. Our results revealed a total of 49 candidate cpSSR loci in the cp genome, and most of these markers were located in the non-coding region, which is consistent with the cp genomes of other angiosperms^57,58^. A previous study reported that the variability of cpSSRs in non-coding regions is more abundant than in conserved coding regions^59^. A strong AT bias, which has been reported in other species^60–62^, also existed in the cp genome of *Rosa* ‘Margo Koster’. Additionally, distribution of cpSSRs was imbalanced in the cp genomes, with over 85% of cpSSRs in the LSC regions.

In addition to repetitive sequences, nucleotide mutation is one of the most important sources of genetic variation. In the comparison of nucleotide diversity among different regions of five cp genomes in *Rosa* species, a set of regions with high divergence was obtained, and most of these regions are intergenic. These highly divergent sequences may be used to develop potential molecular markers for geographical and population genetic studies in *Rosa* spp. Finding polymorphic nucleotide variation in the cp genome used to be time-consuming and labor-intensive work because the available sequences were insufficient. However, highly divergent sequences in *Rosa* spp. have been identified here and will contribute to genetic and evolutionary studies in *Rosa* spp. as well as to wider applications in DNA profiling of rose varieties.

Molecular markers from the cp genome have several advantages, such as a moderate mutant rate, clear genotype, etc. Hence, these nucleotide variations can be used to discriminate varieties including those with close relationships, and to unveil the genetic variations at the population and individual levels. Additionally, the chloroplast SNVs and SSRs could be used to explore the genetic structure and population gene flow^63,64^. Herein, approximately 15 cpSSRs were shown to be polymorphic, although their discriminating power in the test rose varieties is lower, this is likely due to the relatively conserved and haplotype genome of these varieties.

At present, under the framework of the UPOV convention, plant breeder’s rights mainly depend on the distinctness of plant’s phenotypes. Therefore, almost all DUS examinations that require intensive field trials, are based on the morphological characteristics of plants. Collecting a large number of rose varieties in a common database is necessary and prerequisite for DUS examinations; this work requires a great deal of labor, material and land, especially for rose, because of its abundant cultivars and worldwide distribution. Based on the available database, the DUS examiner will select the most similar varieties to start a field trial. Then, according to DUS test guidelines, potential morphological divergences between candidate variety and similar variety will be observed and recorded during at least one growth cycle^23^. For roses, no less than 9 healthy and uniform clones of the candidate variety are required to submit to the national DUS examination station, where these plants will be compared with similar varieties. Over 40 phenotypic traits involved in leaf, flower and fruit, need to be observed and measured during at least one growth cycle. Finally, the examiner will complete a technical questionnaire, which is the most import document to determinate a new variety. The most challenging aspect of this process is the selection of appropriate similar varieties from over ten thousand of existing varieties, and then providing an accurate judgment about the candidate variety in one growth cycle. Because of this challenge, at the end of last century, developing DNA markers for DUS testing, variety profiling or origin tracing was proposed, and case studies in crop, vegetables and fruit trees have been introduced in BMT sessions^65^ or reported in scientific journals^66–68^. For rose, codominant markers from the nuclear genome have been developed and used in genetic studies of diploid and tetraploid rose or DNA profiles^69–73^. Few studies have focused on the genetic variation rose chloroplast genome^74,75^. In this study, we found that there are a large number of highly divergent regions in the rose cp genome, which could provide potential haplotype’ DNA markers. Previous reports also have shown that chloroplast DNA diversity may be a new approach for distinguishing crops, fruits cultivars or tree varieties^76–81^. Our findings showed that the SSRs and SNVs from the cp genome could also distinguish the test rose varieties, although the discrimination power of cpSSR was relatively lower than that of genomic markers. Similar results have been found in *Prunus mume* cultivar and tetraploid alfalfa^82,83^. cpSSR has also been used in individual identification of *Cupressaceae* species and genetic diversity assessment of cultivated and wild *Hevea rubber*^84,85^. We believe that the cpSSRs markers of rose could also be applied to further genetic studies.

In addition to SSR markers, SNPs are a powerful tool for genetic studies in plants, and these markers have been used to evaluate the genetic differentiation among accessions as well as population structure and diversity in crops^86^. A large number of SNP markers have been mined from the nuclear genome of roses^72,87,88^_,_ and an SNP array (WagRhSNP68k) was developed for genetic mapping in tetraploid cut roses^73,89^. Based on the comparative analysis of nucleotide diversity among regions in these *Rosa* species, a set of 20 regions with high divergence have been found and these regions could be used as a starting point for candidate molecular markers for phylogenetic and phylogeographic studies in the *Rosa* genus. Uncovering polymorphic sequences in the cp genome is difficult, particularly when no previous reference genome has been published. The lack of available and polymorphic sequences prevents us to utilize in a phylogenetic context; however, we still expect that the highly divergent sequences identified here by comparing *Rosa* cp genomes will offer new tools for genetic and evolutionary studies in the *Rosa* genus and other related taxa. Our findings indicate that nucleotide mutations in the cp genomes of *Rosa* species are abundant and unambiguous, and the discriminating power of SNVs in the cp genome is stronger than that of cpSSRs in the test rose varieties. Although nuclear SSRs and SNPs have been proven to be reliable and powerful markers for DNA profiling in roses, we also expect that these highly abundant and polymorphic nucleotide variations in the cp genome will become clearly identified, effective and reliable supplementary tools for managing and profiling rose varieties in the future.

## Materials and methods

### Sampling and DNA extraction

All rose varieties used in this study were planted and collected from the National Rose DUS examination station, which is responsible for the DUS testing for rose varieties (Kunming, Yunnan). Fresh leaves were collected from healthy and strong plants, wrapped and stored on dry ice (− 70 °C) until analysis; a list of tested rose varieties is shown in Table S5. *Rosa* ‘Margo Koster’ was used to perform next-generation sequencing to construct the whole cp genome, and other rose varieties were used to test and validate the polymorphism of molecular markers. Genomic DNA was isolated using a DNA extraction Kit (DP-305, Tiangen Biotech, Beijing CO. LTD); agarose gel electrophoresis and a one-drop spectrophotometer were used to detect DNA integrity and quality (Spectramax I3 Microplate Reader, Molecular Devices, USA).

### DNA sequencing, genome assembly, annotation and validation

DNA from *Rosa* ‘Margo Koster’ was used to construct shotgun libraries (250 bp) and sequences on an Illumina HiSeq 2,500 platform. Raw data were trimmed from both ends, individual bases were removed, and entire reads with a median quality score lower than Q20 or less than 25 bp in length after trimming were also discarded. After quality filtering, reads were mapped to an available cp genome of a closely related species (*Rosa roxburghii*; Accession: KX768420.1 GI:1104307301) using Bowtie2 v2.2.6. Then, all putative chloroplast reads mapped to *the Rosa roxburghii* reference above were used for de novo assembly to reconstruct the *Rosa* ‘Margo Koster’ cp genome using SOAPdenovo v2.04 with different kmer sizes^90^. The local gap filling and base correction of contigs were performed by GapCloser v1.12. Finally, in accordance with the size of the contigs, the number of scaffolds and scaffold N50, the best kmer size was used for de novo assembly. Thirty-five primer pairs were used to validate junctions using PCR-based sequencing in *Rosa* ‘Margo Koster’. PCR was performed using a thermal cycler (Applied Biosystems, Foster, CA, USA) with a 20 µL reaction volume as follows: 10 μL of 2 × Taq MasterMix (CWbiotech, Beijing, China), approximately 50 ng of DNA, 5 pmol forward primer, 5 pmol reverse primer, and sterile double-distilled water were added to reach the 20 μL volume. The amplifications were performed using the following schedule: denaturation at 94°C for 5 min; 35 cycles of denaturation for 30 s at 94°C, annealing for 30 s at the optimal temperature, and then extension for 30 s at 72°C; and a final extension at 72°C for 5 min. After PCR amplification, fragments were sequenced and aligned with the assembly cp genome (Table S1). Finally, the corrected cp genome was deposited into GenBank. Predictions of gene, rRNA and tRNA sequences were performed using DOGMA^91^ and manual correction. Gene annotation of *Rosa* ‘Margo Koster’ was obtained from the Nr, KEGG, COG and GO databases using BLAST2.2.28+ based on the predicted protein sequences. A circular representation of the cp genome was drawn using Organellar GenomeDRAW^92^, and the results of annotations were visualized by CGV^93^.

### Repeat sequence analyses in the chloroplast genome

Simple sequence repeats (SSRs) on the cp genome were mined using MISA software (MicroSAtellite, https://pgrc.ipk-gatersleben.de/misa/). The minimal repeat units were set as mono-12, dimer-6, trimer-5, tetramer-5, pentamer-4 and hexamer-4, and Primer 3 was used for designing primer pairs in the flanking region of each candidate locus^94^. Tandem repeat sequences were analyzed by Tandem Repeats Finder^95^, the alignment parameters were set at recommended values (Match-2, Mismatch-7, Delta-7), and the minimum alignment score and maximum period size were set as 80 and 500, respectively. Palindromic repeat sequences, dispersed repeat sequences (including the forward repeats and inverse repeats) and complement repeats were analyzed by REPuter (https://bibiserv.cebitec.uni-bielefeld.de/reputerl)^96^, and the minimum repeat size and maximum base mismatch were set as 30 and 3, respectively.

### Comparative analyses of the chloroplast genome in Rosaceae species

To perform a comparative genomic analysis within the Rosaceae family and *Rosa* genus, 19 species in the Rosaceae family, which are available in the NCBI database, were chosen (Table S4). Then, we used mVISTA software^97^ in shuffle-LANGAN mode and with default parameters for other options to compare the cp genomes from five *Rosa* species, using the sequenced *Rosa roxburghii* annotated genome as a reference. To examine expansion or contraction of the IR regions, boundaries between the four main compositions of the annotated cp genome (LSC, IRa, SSC and IRb) were inspected among 21 species using IRSCOPE software^98^.

To identify regions of high genetic divergence among *Rosa* species that could potentially inform genetic studies of the genus, the genetic divergence among five *Rosa* species across the entire cp genome was calculated using nucleotide diversity (π), and the total number of mutations (η) for gene and intron sequences and intergenic spacers (IGS) was aligned with Verdant and using DnaSP 5.0^99^.

### Phylogenetic analysis

The phylogenetic relationship among the Rosaceae family members was reconstructed using the partial set of species sampled in our studies, including 20 species available in NCBI, one described in our studies, and two species of different orders as outgroups (*Amborella trichopoda* and *Nymphaea alba*). First, complete cp genome sequences were aligned using MAFFT software, and then maximum likelihood (ML) and neighbor-joining (NJ) were used to reconstruct the phylogenetic tree with 2000 bootstrap replicates using MEGA 7.0 software^100^.

### Polymorphism validation of candidate molecular markers

To verify the polymorphism of candidate cpSSRs and SNVs, genotyping PCR was performed using a thermal cycler (Applied Biosystems, Foster, CA, USA) with a 20 µL reaction volume as follows: 10 μL of 2 × Taq MasterMix (CWbiotech, Beijing, China), approximately 50 ng of DNA, 5 pmol forward primer, 5 pmol reverse primer, and sterile double-distilled water were added to reach the 20 μL volume. The amplifications were performed using the following schedule: denaturation at 94°C for 5 min; 35 cycles of denaturation for 30 s at 94°C, annealing for 30 s at the optimal temperature, and then extension for 30 s at 72°C; and a final extension at 72°C for 5 min. The PCR products were purified, analyzed by capillary electrophoresis and sequenced by Sanger’s method. Finally, polymorphisms of candidate cpSSRs and SNVs from flanking regions were aligned and analyzed using MEGA^100^ and DNASP5.0^99^. D_j_ is the value of assessing the discriminating power of molecular marker^101^, and is calculated as $$D_{j} = 1 - C_{j} = 1 - \sum\nolimits_{i = 1}^{i} {p_{i} {\raise0.7ex\hbox{${Np_{i} - 1}$} \!\mathord{\left/ {\vphantom {{Np_{i} - 1} {N - 1}}}\right.\kern-\nulldelimiterspace} \!\lower0.7ex\hbox{${N - 1}$}}}$$.

## Supplementary information

Supplementary information
