# Supplementary material for: Molecular markers from the chloroplast genome of rose provide a complementary tool for variety discrimination and profiling
Source: Sci Rep. 2020 Jul 22;10:12188. doi: 10.1038/s41598-020-68092-1 (PMC7376030; doi:10.1038/s41598-020-68092-1)
Supplement: Supplementary file 1 — Supplementary information [file 41598_2020_68092_MOESM1_ESM.docx]

**Molecular markers from the chloroplast genome of rose provide new complementary tool for variety discrimination and profile**

Li Changhong, Zheng Yongqi^*^, Huang Ping^*^

State Key Laboratory of Tree Genetics and Breeding; Key Laboratory of Forest Silviculture and Tree Cultivation, State Forestry Administration; Research Institute of Forestry, Chinese Academy of Forestry, Beijing 100091, China.

*****Correspondence: [zhengyq@caf.ac.cn](mailto:zhengyq@caf.ac.cn);Tel: +86-10-62888565; [huangping@caf.ac.cn](mailto:huangping@caf.ac.cn); Tel.: +86-10-62889592


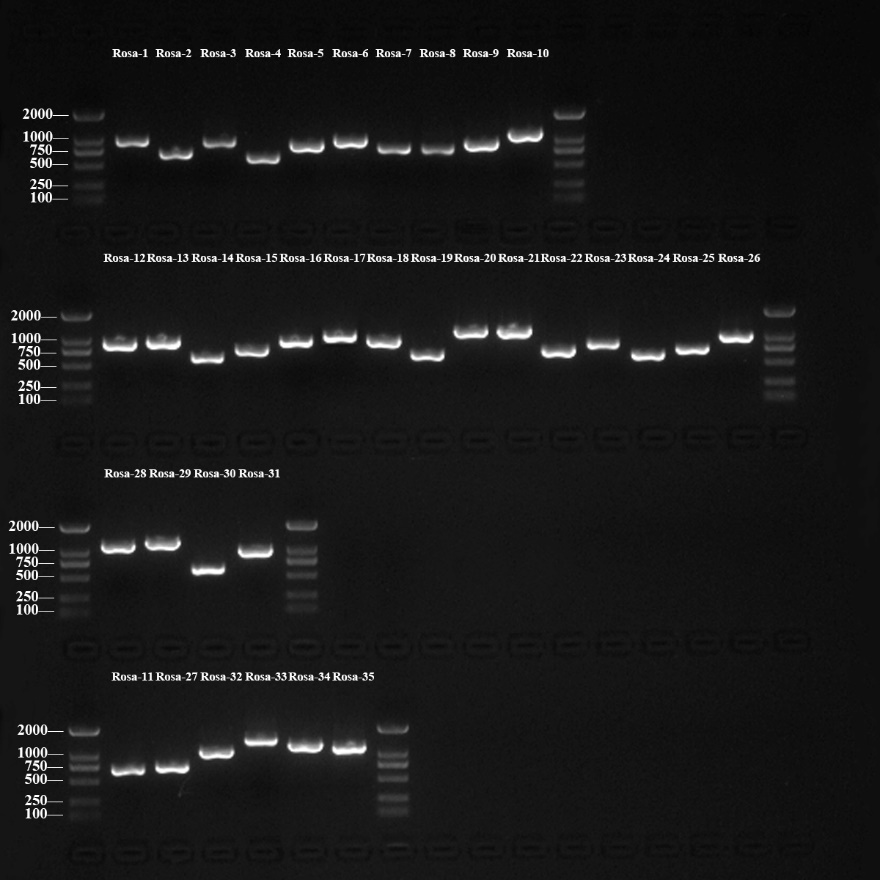


**Figure S1** The gel photo of PCR products for validating and correcting chloroplast genome sequences base on NGS assembly. The PCR products were separated on 1.5% agarose gels, and the D2000 DNA ladder were loaded as markers. All the PCR products have been sequenced by Sanger’s method, and these regions in cp genome also have been aligned and corrected base on sanger’s sequence results, five sequences of them are across four conjunction regions e.g., *Rosa32* (LSC-IRb), *Rosa33* (IRb-SSC), *Rosa34* (SSC-IRa) and *Rosa35* (LSC-IRa).


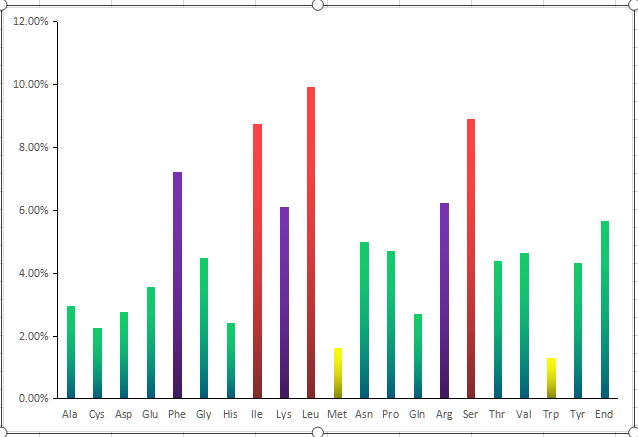


**Figure S2.** Frequency of amino acids in the cp genome of *Rosa* ‘Margo Koster’

**
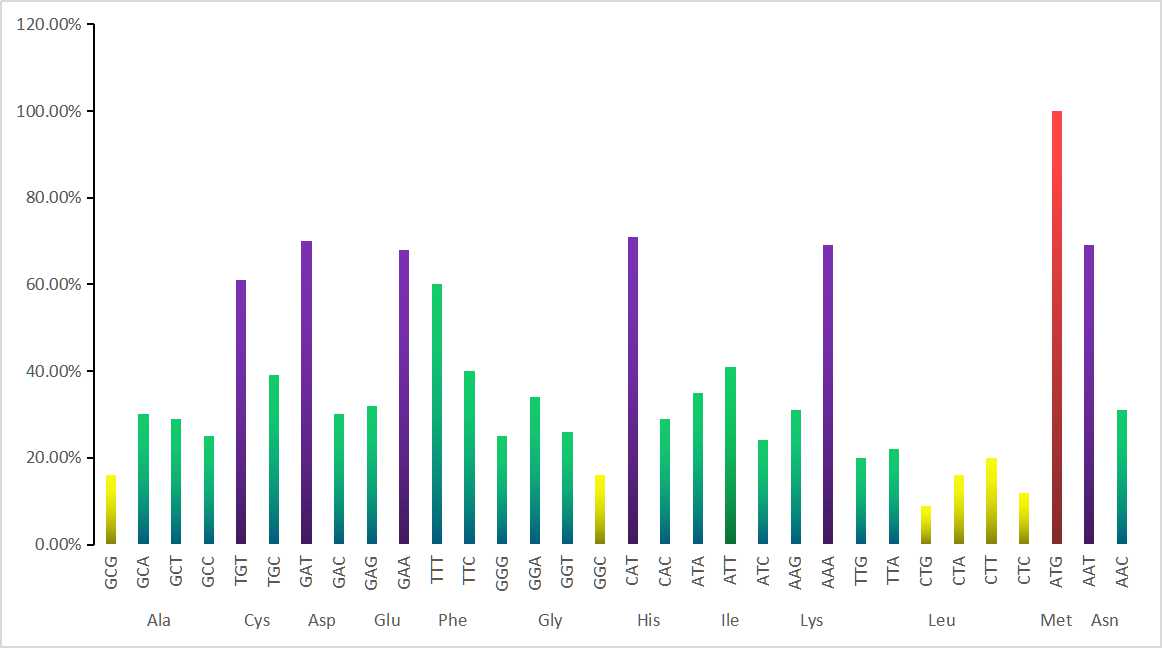
**

**Figure S3-1.** Codon usage in the cp genome of *Rosa* ‘Margo Koster’

**
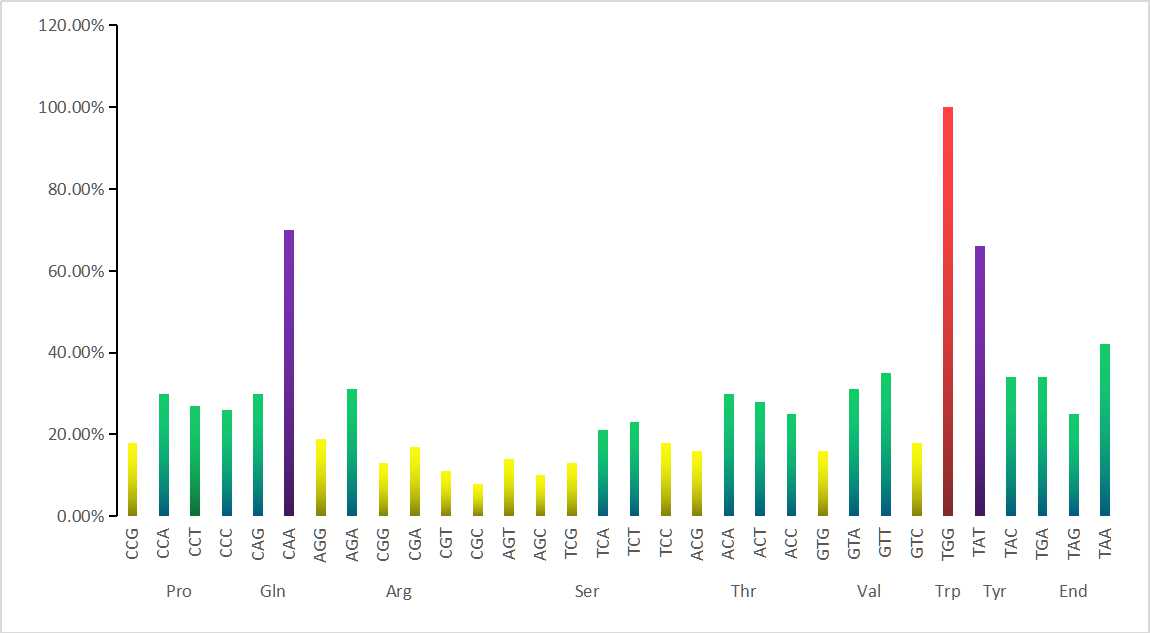
**

**Figure S3-2.** Codon usage in the cp genome of *Rosa* ‘Margo Koster’

**
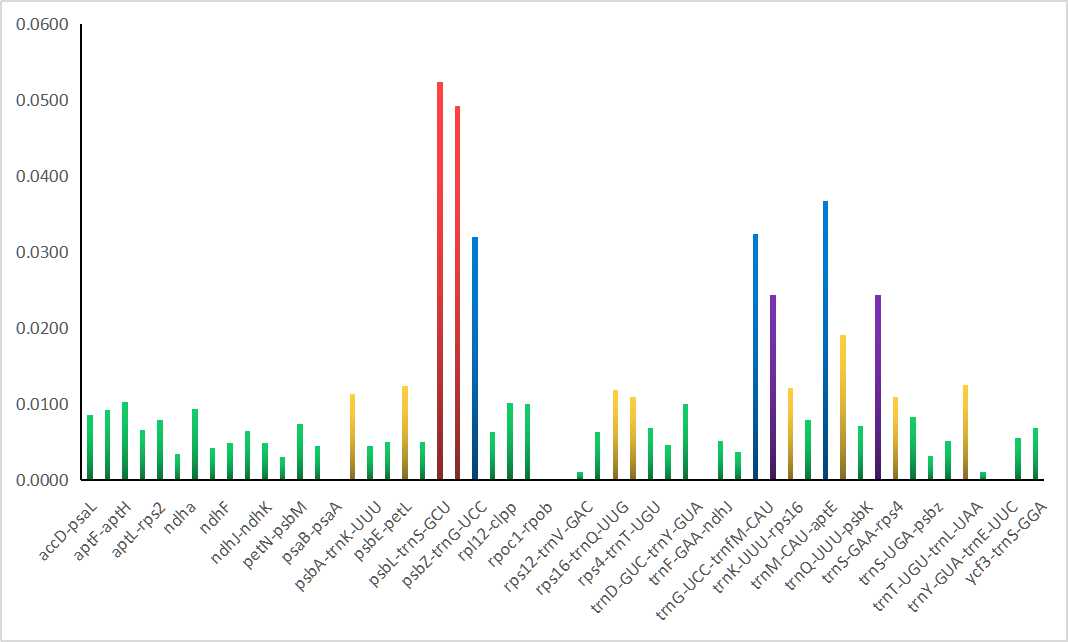
**

**Figure S4.** Nucleotide diversity (π) of sequence regions in the cp genome among five *Rosa* species

**Table S1 Results of PCR-based verification of the cp genome and primer information**

| **Name** | **Forward primer** | **Reverse primer** | **Purification** | **Tm**  **（℃）** | **Size of validated sequence*（bp）** | **Validated region** |
| --- | --- | --- | --- | --- | --- | --- |
| Rosa-1 | GGTAAAGGAGCAACACCAAAC | CTGGTGCCATTATCCCTACTT | PAGE | 56 | 847 | LSC (353-1199) |
| Rosa-2 | CGTATATAGCTAGAACGACCCTCAC | TGGAAGAGATGATGGAAGCAG | PAGE | 58 | 593 | LSC (15616-16208) |
| Rosa-3 | GGTTGAAGTAGTTGAATAGGAGG | ACTCCAAGAGCACTCATCCATA | PAGE | 57 | 847 | LSC (33671-34517) |
| Rosa-4 | CCACTGATAAACACCAGAATAAGC | AATGTTTCTATTCAGCGGACG | PAGE | 57 | 508 | LSC (40296-40903) |
| Rosa-5 | GACATCACATTGATAGCCTCGAC | CGTACTATCAAGAGGATTAGCTGC | PAGE | 59 | 813 | LSC (54407-55219) |
| Rosa-6 | GTGTTGGATTCAAAGCTGGTG | GGATATGATCTCCACCAGACATAC | PAGE | 60 | 891 | LSC (57120-58010) |
| Rosa-7 | TCTATCTATAGTCATTGGGGCCTC | CTATTAGTACTGGGCGACTAACCA | PAGE | 59 | 695 | LSC (66102-66796） |
| Rosa-8 | GTTCCTTTCTTCTTCCTCGTTTC | TTCTAAGACCAGTAGTTCTAGCGGT | PAGE | 58 | 680 | LSC (69388-70067) |
| Rosa-9 | GTCGCACTATACGTCAATCCAC | GATCCCAATACACCCAATGC | PAGE | 57 | 846 | LSC (73814-74659) |
| Rosa-10 | CAGGGTTCATAACTACTCCTCTTAC | ATACGACCCTAATCGAAATGC | PAGE | 57 | 974 | LSC (86994-87592) |
| Rosa-11 | CCACACCAATTCGTTATGTATGG | GATACAGGAGCGAAACAATCAAC | PAGE | 57 | 598 | IRb (88380-88977) |
| Rosa-12 | CATACCAAATCCCATCAATCG | GAACATCTTTCACAATCCCTGG | PAGE | 57 | 857 | IRb (92972-93828) |
| Rosa-13 | ACGAATCGGAGTTTGAAGAAG | GTGAGTAATAGCTCTGGTTGTTCG | PAGE | 56 | 857 | IRb (94743-95599) |
| Rosa-14 | GAGAAGATCACCAAGATTTCGTG | AGAATTAGTAGATCTGTTCCGCC | PAGE | 56 | 528 | IRb (96344-96871) |
| Rosa-15 | CGGATAGAGGAATACATAGAGTTG | AAGTTCCGAATTAGTGGATGC | PAGE | 57 | 677 | IRb (98868-99544) |
| Rosa-16 | GATAGGAAAGATCTCCCTCCAAG | GTGACTGAATTGGTCCTGGTTAC | PAGE | 56 | 855 | IRb (10031-100885) |
| Rosa-17 | CGAATGAGAATGGATAAGAGGC | GCATGATGACTTGACGTCATC | PAGE | 59 | 955 | IRb (102702-103655） |
| Rosa-18 | GTCTGGAGAAAGCTGCAATCA | AGGGAGATAGTGCATCAAGCTG | PAGE | 56 | 867 | IRb (105112-105978) |
| Rosa-19 | CGATTACGGGTTGGATGTCTA | GCTGAGTTGGAATCCCATTCT | PAGE | 57 | 570 | IRb (105811-106380) |
| Rosa-20 | TTGTCTATCGTCGGCCTCTATG | CAGAGTTTGCCTCGATTTGGT | PAGE | 56 | 1074 | IRb (106567-107640) |
| Rosa-21 | CAAGGAAGTTGGTGACCTGATG | ATGGCGAGTGCCTGATCAAT | PAGE | 58 | 1077 | IRb (108681-109757) |
| Rosa-22 | AGGGAATCTAGTATCCTTATGCATG | CTTAGTAACAATGGGTGACGGTA | PAGE | 58 | 645 | IRb (111440-112084) |
| Rosa-23 | CTATGAAAAGAGGAAGAAGCCG | TCATACCGTTCATTCCACTTCC | PAGE | 57 | 799 | SSC (113944-114713) |
| Rosa-24 | CGTAAGTATGATGGTATTGGGCT | GGTTCTTGATGGTTTACATGAGG | PAGE | 57 | 548 | SSC (117448-117995) |
| Rosa-25 | GACCCATACTTCGAGTTGTTTCA | ACGAGCCGAAATATGGTTAGAG | PAGE | 57 | 632 | SSC (119896-120527) |
| Rosa-26 | ACATCGGTAAACGACCCAAAG | GCGTCAGCCTATAGGGTTTATC | PAGE | 57 | 828 | SSC (121911-122738) |
| Rosa-27 | GTTTCTTAGTAACAATGGGTGACG | GGGAATCTAGTATCCTTATGCATG | PAGE | 58 | 635 | SSC-IRa (131713-132347) |
| Rosa-28 | CAATGAAAATAGATGGCGAGTG | CGTAACTATAACGGTCCTAAGGTA | PAGE | 57 | 1037 | IRa (133955-134991) |
| Rosa-29 | TTAAGGTAACGACTTCGGGC | TAATACCCCGTAGGCTGAGGA | PAGE | 57 | 1149 | IRa (139532-140680) |
| Rosa-30 | CAGAGACAAAATGTAGGACTGGTG | GACCCATTTTCTCATTAAGCG | PAGE | 57 | 520 | IRa (142096-142615) |
| Rosa-31 | ACTCCGGATATAGCAGTAAAAGC | CCTCCTAGAGTAGCTGTTAATACGA | PAGE | 58 | 877 | IRa (144038-144914) |
| Rosa-32 | GGAATCAAAATCTGCCGAA | AGCTATGAATAGTCATCGACTCC | PAGE | 58 | 963 | LSC-IRb (82064-88026) |
| Rosa-33 | GGAAAGTGAGGAAGAAACAGATG | CTCTTGCTTGTTTTTGGTCCA | PAGE | 58 | 1281 | IRb-SSC (113444-112164) |
| Rosa-34 | TAGAGGGGACTTTTTCCTTTGG | TGGATTTCTGACCACATTCTCC | PAGE | 58 | 1101 | SSC-IRa (131155-1332255) |
| Rosa-35 | AGGAACGGGAAGACCTAGGAT | CTCTAGACCTAGCTGCTGTTGAAG | PAGE | 58 | 1038 | LSC-IRa (156656-398) |

* the size of validated sequence is the number of base pair which can be mapped to reference cp genome

**Table S2. Codon-anticodon recognition patterns and codon usage of *Rosa* ‘Margo Koster’ cp genome**

| **AmAcid** | **Codon** | **Number** | **CBI** | **AmAcid** | **Codon** | **Number** | **CBI** | **AmAcid** | **Codon** | **Number** | **CBI** |
| --- | --- | --- | --- | --- | --- | --- | --- | --- | --- | --- | --- |
| Ala | GCT | 456 | 1.17 | Lys | AAG | 996 | 0.62 | Ser | TCT | 1082 | 1.39 |
| Ala | GCC | 385 | 0.99 | Leu | TTA | 1160 | 1.33 | Ser | TCC | 849 | 1.09 |
| Ala | GCA | 461 | 1.19 | Leu | TTG | 1067 | 1.23 | Ser | TCA | 1001 | 1.28 |
| Ala | GCG | 254 | 0.65 | Leu | CTT | 1026 | 1.18 | Ser | TCG | 598 | 0.77 |
| Cys | TGT | 732 | 1.23 | Leu | CTC | 649 | 0.75 | Ser | AGT | 663 | 0.85 |
| Cys | TGC | 461 | 0.77 | Leu | CTA | 834 | 0.96 | Ser | AGC | 486 | 0.62 |
| Asp | GAT | 1024 | 1.41 | Leu | CTG | 479 | 0.55 | Thr | ACT | 653 | 1.14 |
| Asp | GAC | 429 | 0.59 | Met | ATG | 845 | 1 | Thr | ACC | 582 | 1.01 |
| Glu | GAA | 1284 | 1.37 | Asn | AAT | 1821 | 1.39 | Thr | ACA | 692 | 1.2 |
| Glu | GAG | 591 | 0.63 | Asn | AAC | 807 | 0.61 | Thr | ACG | 372 | 0.65 |
| Phe | TTT | 2283 | 1.2 | Pro | CCT | 666 | 1.08 | Val | GTT | 851 | 1.39 |
| Phe | TTC | 1512 | 0.8 | Pro | CCC | 640 | 1.04 | Val | GTC | 443 | 0.72 |
| Gly | GGT | 598 | 1.02 | Pro | CCA | 731 | 1.18 | Val | GTA | 753 | 1.23 |
| Gly | GGC | 366 | 0.62 | Pro | CCG | 434 | 0.7 | Val | GTG | 399 | 0.65 |
| Gly | GGA | 801 | 1.37 | Gln | CAA | 995 | 1.4 | Trp | TGG | 677 | 1 |
| Gly | GGG | 578 | 0.99 | Gln | CAG | 426 | 0.6 | Tyr | TAT | 1503 | 1.33 |
| His | CAT | 900 | 1.42 | Arg | CGT | 371 | 0.68 | Tyr | TAC | 761 | 0.67 |
| His | CAC | 368 | 0.58 | Arg | CGC | 265 | 0.49 | TER | TAA | 1232 | 1.25 |
| Ile | ATT | 1875 | 1.23 | Arg | CGA | 569 | 1.04 | TER | TAG | 730 | 0.74 |
| Ile | ATC | 1121 | 0.73 | Arg | CGG | 411 | 0.75 | TER | TGA | 1002 | 1.01 |
| Ile | ATA | 1595 | 1.04 | Arg | AGA | 1018 | 1.87 |  |  |  |  |
| Lys | AAA | 2217 | 1.38 | Arg | AGG | 635 | 1.17 |  |  |  |  |

CBI: Codon Bias index

**Table S3.** Summary of complete cp genome features of 19 species within the Rosaceae family and 2 species of basal angiosperms

| **No.** | **Species** | **GenBank**  **numbers** | **Genus** | **No. rRNA** | **No.**  **tRNA** | **Protein -coding genes** | **No.**  **genes** | **Genome Size（bp）** | **LSC length（bp）** | **IR length（bp）** | **SSC length（bp）** |
| --- | --- | --- | --- | --- | --- | --- | --- | --- | --- | --- | --- |
| 1 | *Rosa* ‘Margo Koster’ | MN435990 | *Rosa* | 8 | 37 | 88 | 133 | 157395 | 87710 | 25418 | 18849 |
| 2 | *Rosa praelucens* | MG450565.1 | *Rosa* | 8 | 37 | 84 | 129 | 157186 | 86313 | 26054 | 18765 |
| 3 | *Rosa chinensis* var. spontanea | NC_038102.1 | *Rosa* | 8 | 37 | 84 | 129 | 156590 | 85910 | 25959 | 18762 |
| 4 | *Rosa roxburghii* | NC_032038.1 | *Rosa* | 8 | 39 | 88 | 135 | 156749 | 85852 | 26053 | 18791 |
| 5 | *Rosa odorata* var. gigantea | KF753637.1 | *Rosa* | 8 | 40 | 88 | 136 | 156634 | 85767 | 26053 | 18761 |
| 6 | *Prunus padus* | KP760072.1 | *Padus* | 8 | 37 | 84 | 129 | 158955 | 87666 | 26209 | 18871 |
| 7 | *Chaenomeles sinensis* | KT932967.1 | *Chaenomeles* | 8 | 36 | 83 | 127 | 159351 | 87463 | 26357 | 19174 |
| 8 | *Hagenia abyssinica* | KX008604.2 | *Hagenia* | 8 | 37 | 85 | 130 | 154961 | 84318 | 25973 | 18697 |
| 9 | *Cydonia oblonga* | KX499857.1 | *Cydonia* | 8 | 37 | 82 | 127 | 159609 | 87823 | 26253 | 19280 |
| 10 | *Malus baccata* | KX499859.1 | *Malus* | 8 | 35 | 82 | 125 | 160163 | 88267 | 26354 | 19188 |
| 11 | *Docynia delavayi* | KX499860.1 | *Docynia* | 8 | 35 | 83 | 126 | 159428 | 87471 | 26357 | 19243 |
| 12 | *Pyrus pyrifolia* | NC_015996.1 | *Pyrus* | 8 | 36 | 83 | 127 | 159922 | 87901 | 26392 | 19237 |
| 13 | *Prunus cerasoides* | NC_035891.1 | *Cerasus* | 8 | 37 | 84 | 129 | 157685 | 85792 | 26416 | 19061 |
| 14 | *Prunus persica* | NC_014697.1 | *Prunus* | 8 | 37 | 85 | 130 | 157790 | 85968 | 26381 | 19060 |
| 15 | *Fragaria vesca subsp.* vescat | NC_015206.1 | *Fragaria* | 8 | 37 | 85 | 130 | 155691 | 85606 | 25956 | 18173 |
| 16 | *Pentactina rupicola* | NC_016921.1 | *Spiraea* | 8 | 37 | 84 | 129 | 156612 | 84970 | 26351 | 18940 |
| 17 | *Sorbus torminalis* | NC_033975.1 | *Sorbus* | 8 | 36 | 83 | 127 | 160390 | 88250 | 26,416 | 19208 |
| 18 | *Chaenomeles japonica* | NC_035566.1 | *Chaenomeles* | 8 | 35 | 83 | 126 | 160088 | 88052 | 26316 | 19404 |
| 19 | *Eriobotrya japonicat* | NC_034639.1 | *Eriobotrya* | 8 | 37 | 84 | 129 | 159137 | 87202 | 26326 | 19283 |
| 20 | *Nymphaea alba* | NC_006050.1 | *Nymphaea* | 8 | 37 | 85 | 130 | 159930 | 90014 | 25177 | 19562 |
| 21 | *Amborella trichopoda* | NC_005086.1 | *Amborella* | 8 | 36 | 84 | 128 | 162686 | 90970 | 26651 | 18414 |

**Table S4-1. Repeat sequences in the cp genome of *Rosa* ‘Margo Koster’**

| **ID** | **Repeat Start 1** | **Type** | **Size(bp)** | **Repeat Start 2** | **Mismatch (bp)** | **E-Value** | **Region** |
| --- | --- | --- | --- | --- | --- | --- | --- |
| 1 | 4988 | F | 181 | 5176 | 0 | 7.42E-100 | LSC |
| 2 | 8471 | F | 32 | 36347 | -3 | 5.06E-05 | LSC |
| 3 | 9908 | F | 30 | 37491 | -3 | 6.62E-04 | LSC |
| 4 | 12290 | F | 30 | 72354 | -3 | 6.62E-04 | LSC |
| 5 | 16389 | F | 31 | 16417 | 0 | 1.51E-09 | LSC |
| 6 | 27756 | F | 31 | 27780 | -3 | 1.83E-04 | LSC |
| 7 | 33285 | F | 55 | 33421 | 0 | 5.37E-24 | LSC |
| 8 | 36971 | F | 34 | 37014 | 0 | 2.36E-11 | LSC |
| 9 | 37338 | F | 50 | 37379 | 0 | 5.50E-21 | LSC |
| 10 | 39745 | F | 30 | 41969 | -3 | 6.62E-04 | LSC |
| 11 | 44414 | F | 33 | 44459 | -1 | 9.35E-09 | LSC |
| 12 | 44805 | F | 39 | 100797 | 0 | 2.31E-14 | LSC |
| 13 | 44805 | F | 38 | 122917 | 0 | 9.22E-14 | LSC |
| 14 | 54277 | F | 38 | 54315 | 0 | 9.22E-14 | LSC |
| 15 | 59071 | F | 34 | 59102 | 0 | 2.36E-11 | LSC |
| 16 | 86971 | F | 33 | 87022 | 0 | 9.44E-11 | LSC |
| 17 | 91145 | F | 32 | 91166 | -3 | 5.06E-05 | IRb |
| 18 | 98165 | F | 31 | 145495 | -3 | 1.83E-04 | IRb |
| 19 | 100795 | F | 40 | 122915 | 0 | 5.76E-15 | IRb |
| 20 | 109823 | F | 30 | 109855 | -2 | 2.37E-05 | IRb |
| 21 | 123062 | F | 70 | 123127 | -1 | 1.05E-30 | SSC |
| 22 | 133835 | F | 30 | 133867 | -2 | 2.37E-05 | IRa |
| 23 | 152493 | F | 32 | 152514 | -3 | 5.06E-05 | IRa |
| 24 | 156800 | F | 32 | 156933 | -3 | 5.06E-05 | IRa |
| 25 | 156844 | F | 36 | 156923 | -2 | 8.37E-09 | IRa |
| 26 | 156853 | F | 30 | 156932 | -3 | 6.62E-04 | IRa |
| 27 | 37409 | R | 35 | 37409 | -2 | 3.16E-08 | LSC |
| 28 | 37417 | R | 31 | 37417 | -2 | 6.32E-06 | LSC |
| 29 | 69635 | R | 33 | 69635 | -2 | 4.49E-07 | LSC |
| 30 | 79787 | R | 31 | 79787 | -2 | 6.32E-06 | LSC |
| 31 | 0 | P | 54 | 86265 | 0 | 2.15E-23 | LSC |
| 32 | 8473 | P | 30 | 46567 | 0 | 6.04E-09 | LSC |
| 33 | 10275 | P | 54 | 10275 | 0 | 2.15E-23 | LSC |
| 34 | 36349 | P | 30 | 46567 | -3 | 6.62E-04 | LSC |
| 35 | 37418 | P | 30 | 37418 | -2 | 2.37E-05 | LSC |
| 36 | 44805 | P | 39 | 142855 | 0 | 2.31E-14 | LSC |
| 37 | 54269 | P | 46 | 54320 | -1 | 1.94E-16 | LSC |
| 38 | 54282 | P | 33 | 54282 | -1 | 9.35E-09 | LSC |
| 39 | 54299 | P | 37 | 54299 | -3 | 7.74E-08 | LSC |
| 40 | 54315 | P | 43 | 54315 | -1 | 1.16E-14 | LSC |
| 41 | 76170 | P | 38 | 76170 | -2 | 5.83E-10 | LSC |
| 42 | 86318 | P | 83 | 157312 | 0 | 7.45E-41 | LSC |
| 43 | 86415 | P | 407 | 156894 | 0 | 6.38E-236 | LSC |
| 44 | 86751 | P | 32 | 156800 | -3 | 5.06E-05 | LSC |
| 45 | 86757 | P | 36 | 156844 | -2 | 8.37E-09 | LSC |
| 46 | 86971 | P | 33 | 156636 | 0 | 9.44E-11 | LSC |
| 47 | 87003 | P | 14991 | 141697 | 0 | 0 0.00e+00 | LSC |
| 48 | 91145 | P | 32 | 152493 | -3 | 5.06E-05 | IRb |
| 49 | 91166 | P | 32 | 152514 | -3 | 5.06E-05 | IRb |
| 50 | 98165 | P | 31 | 98165 | -3 | 1.83E-04 | IRb |
| 51 | 101992 | P | 4426 | 137271 | 0 | 0 0.00e+00 | IRb |
| 52 | 106439 | P | 6009 | 131272 | 0 | 0 0.00e+00 | IRb |
| 53 | 109823 | P | 30 | 133835 | -2 | 2.37E-05 | IRb |
| 54 | 109855 | P | 30 | 133867 | -2 | 2.37E-05 | IRb |
| 55 | 122915 | P | 40 | 142856 | 0 | 5.76E-15 | SSC |
| 56 | 145495 | P | 31 | 145495 | -3 | 1.83E-04 | IRa |
| 57 | 6569 | C | 31 | 61070 | -3 | 1.83E-04 | LSC |

F: forward repeat, C: complement repeat, R: reverse repeat, P: palindromic repeat.

**Table S4-2.** Repeat sequences in the cp genome of *Rosa* ‘Margo Koster’

|  | **Indices** | **Type** | **Period Size** | **Period Size** | **Period Size** | **Period Size** | **Period Size** | **Score** | **A** | **C** | **G** | **T** | **Entropy (0-2)** | **Region** |
| --- | --- | --- | --- | --- | --- | --- | --- | --- | --- | --- | --- | --- | --- | --- |
| 58 | [4982--5357](http://tandem.bu.edu/trf/output/13WRcUhzbPbNU.2.7.7.80.10.80.500.1.txt.html#4982--5357,188,2.0,187,1) | T | 188 | 2 | 187 | 99 | 0 | 743 | 23 | 17 | 13 | 46 | 1.83 | LSC |
| 59 | [10591--10631](http://tandem.bu.edu/trf/output/13WRcUhzbPbNU.2.7.7.80.10.80.500.1.txt.html#10591--10631,19,2.2,19,2) | T | 19 | 2.2 | 19 | 100 | 0 | 82 | 29 | 0 | 4 | 65 | 1.13 | LSC |
| 60 | [16390--16448](http://tandem.bu.edu/trf/output/13WRcUhzbPbNU.2.7.7.80.10.80.500.1.txt.html#16390--16448,28,2.1,28,3) | T | 28 | 2.1 | 28 | 100 | 0 | 118 | 22 | 23 | 6 | 47 | 1.75 | LSC |
| 61 | [27764--27811](http://tandem.bu.edu/trf/output/13WRcUhzbPbNU.2.7.7.80.10.80.500.1.txt.html#27764--27811,24,2.0,24,4) | T | 24 | 2 | 24 | 100 | 0 | 96 | 41 | 12 | 8 | 37 | 1.73 | LSC |
| 62 | [28679--28718](http://tandem.bu.edu/trf/output/13WRcUhzbPbNU.2.7.7.80.10.80.500.1.txt.html#28679--28718,20,2.0,20,5) | T | 20 | 2 | 20 | 100 | 0 | 80 | 30 | 10 | 5 | 55 | 1.54 | LSC |
| 63 | [33286--33554](http://tandem.bu.edu/trf/output/13WRcUhzbPbNU.2.7.7.80.10.80.500.1.txt.html#33286--33554,136,1.9,135,6) | T | 136 | 1.9 | 135 | 89 | 4 | 403 | 33 | 13 | 20 | 31 | 1.92 | LSC |
| 64 | [36966--37054](http://tandem.bu.edu/trf/output/13WRcUhzbPbNU.2.7.7.80.10.80.500.1.txt.html#36966--37054,43,2.1,43,7) | T | 43 | 2.1 | 43 | 95 | 4 | 162 | 13 | 20 | 19 | 47 | 1.82 | LSC |
| 65 | [37342--37408](http://tandem.bu.edu/trf/output/13WRcUhzbPbNU.2.7.7.80.10.80.500.1.txt.html#37342--37408,20,3.3,20,8) | T | 20 | 3.3 | 20 | 76 | 12 | 82 | 58 | 2 | 2 | 35 | 1.29 | LSC |
| 66 | [37355--37448](http://tandem.bu.edu/trf/output/13WRcUhzbPbNU.2.7.7.80.10.80.500.1.txt.html#37355--37448,12,7.3,12,9) | T | 12 | 7.3 | 12 | 81 | 13 | 98 | 58 | 1 | 1 | 39 | 1.12 | LSC |
| 67 | [37332--37429](http://tandem.bu.edu/trf/output/13WRcUhzbPbNU.2.7.7.80.10.80.500.1.txt.html#37332--37429,41,2.4,41,10) | T | 41 | 2.4 | 41 | 98 | 1 | 189 | 58 | 2 | 2 | 37 | 1.21 | LSC |
| 68 | [44405--44526](http://tandem.bu.edu/trf/output/13WRcUhzbPbNU.2.7.7.80.10.80.500.1.txt.html#44405--44526,45,2.6,46,11) | T | 45 | 2.6 | 46 | 90 | 5 | 183 | 17 | 15 | 5 | 61 | 1.52 | LSC |
| 69 | [54278--54353](http://tandem.bu.edu/trf/output/13WRcUhzbPbNU.2.7.7.80.10.80.500.1.txt.html#54278--54353,38,2.0,38,12) | T | 38 | 2 | 38 | 100 | 0 | 152 | 39 | 13 | 15 | 31 | 1.86 | LSC |
| 70 | [59072--59136](http://tandem.bu.edu/trf/output/13WRcUhzbPbNU.2.7.7.80.10.80.500.1.txt.html#59072--59136,31,2.1,31,13) | T | 31 | 2.1 | 31 | 100 | 0 | 130 | 30 | 12 | 9 | 47 | 1.72 | LSC |
| 71 | [60692--60786](http://tandem.bu.edu/trf/output/13WRcUhzbPbNU.2.7.7.80.10.80.500.1.txt.html#60692--60786,42,2.2,44,15) | T | 42 | 2.2 | 44 | 85 | 11 | 124 | 55 | 2 | 8 | 33 | 1.42 | LSC |
| 72 | [67379--67426](http://tandem.bu.edu/trf/output/13WRcUhzbPbNU.2.7.7.80.10.80.500.1.txt.html#67379--67426,19,2.6,19,17) | T | 19 | 2.6 | 19 | 96 | 3 | 89 | 25 | 20 | 10 | 43 | 1.83 | LSC |
| 73 | [68490--68529](http://tandem.bu.edu/trf/output/13WRcUhzbPbNU.2.7.7.80.10.80.500.1.txt.html#68490--68529,19,2.1,19,18) | T |  | 2.1 | 19 | 100 | 0 | 80 | 27 | 10 | 0 | 62 | 1.27 | LSC |
| 74 | [86947--87000](http://tandem.bu.edu/trf/output/13WRcUhzbPbNU.2.7.7.80.10.80.500.1.txt.html#86947--87000,25,2.1,27,19) | T | 25 | 2.1 | 27 | 93 | 6 | 94 | 37 | 22 | 11 | 29 | 1.89 | LSC |
| 75 | [91146--91213](http://tandem.bu.edu/trf/output/13WRcUhzbPbNU.2.7.7.80.10.80.500.1.txt.html#91146--91213,21,3.2,21,21) | T | 21 | 3.2 | 21 | 82 | 0 | 82 | 11 | 22 | 7 | 58 | 1.57 | IRb |
| 76 | [101551--101609](http://tandem.bu.edu/trf/output/13WRcUhzbPbNU.2.7.7.80.10.80.500.1.txt.html#101551--101609,11,5.5,11,22) | T | 11 | 5.5 | 11 | 90 | 6 | 95 | 33 | 1 | 16 | 47 | 1.57 | IRb |
| 77 | [101548--101609](http://tandem.bu.edu/trf/output/13WRcUhzbPbNU.2.7.7.80.10.80.500.1.txt.html#101548--101609,21,3.0,21,23) | T | 21 | 3 | 21 | 92 | 4 | 99 | 32 | 3 | 16 | 48 | 1.62 | IRb |
| 78 | [106391--106449](http://tandem.bu.edu/trf/output/13WRcUhzbPbNU.2.7.7.80.10.80.500.1.txt.html#106391--106449,27,2.0,29,24) | T | 27 | 2 | 29 | 87 | 12 | 86 | 23 | 5 | 23 | 47 | 1.71 | IRb |
| 79 | [109824--109885](http://tandem.bu.edu/trf/output/13WRcUhzbPbNU.2.7.7.80.10.80.500.1.txt.html#109824--109885,32,1.9,32,27) | T | 32 | 1.9 | 32 | 93 | 0 | 106 | 38 | 22 | 11 | 27 | 1.88 | IRb |
| 80 | [123063--123197](http://tandem.bu.edu/trf/output/13WRcUhzbPbNU.2.7.7.80.10.80.500.1.txt.html#123063--123197,65,2.1,65,28) | T | 65 | 2.1 | 65 | 98 | 0 | 261 | 40 | 3 | 7 | 48 | 1.49 | SSC |
| 81 | [133836--133897](http://tandem.bu.edu/trf/output/13WRcUhzbPbNU.2.7.7.80.10.80.500.1.txt.html#133836--133897,32,1.9,32,30) | T | 32 | 1.9 | 32 | 93 | 0 | 106 | 27 | 11 | 22 | 38 | 1.88 | IRa |
| 82 | [142083--142141](http://tandem.bu.edu/trf/output/13WRcUhzbPbNU.2.7.7.80.10.80.500.1.txt.html#142083--142141,11,5.5,11,31) | T | 11 | 5.5 | 11 | 90 | 6 | 95 | 47 | 16 | 1 | 33 | 1.57 | IRa |
| 83 | [142083--142144](http://tandem.bu.edu/trf/output/13WRcUhzbPbNU.2.7.7.80.10.80.500.1.txt.html#142083--142144,21,3.0,21,32) | T | 21 | 3 | 21 | 92 | 4 | 99 | 48 | 16 | 3 | 32 | 1.62 | IRa |
| 84 | [152480--152568](http://tandem.bu.edu/trf/output/13WRcUhzbPbNU.2.7.7.80.10.80.500.1.txt.html#152480--152568,21,4.1,21,33) | T | 21 | 4.1 | 21 | 81 | 5 | 81 | 56 | 7 | 23 | 12 | 1.62 | IRa |

T: tandem repeats

**Table S5.** Rose varieties used to evaluate the polymorphism and discriminating power

| **No.** | **Name** | **No.** | **Name** | **No.** | **Name** |
| --- | --- | --- | --- | --- | --- |
| A01 | Grand Gala | A56 | Queen Elizabeth | A216 | Rigoletto |
| A02 | First Red | A58 | Hojun | A219 | Revival |
| A03 | Charlotte | A60 | Mu Guafen | A221 | Guy de Maupassants |
| A04 | Sangria' 90 | A72 | Blue Ribbon | A224 | Scepter’d Isle |
| A08 | Red France | A81 | Frisco | A226 | St. Ethelburga |
| A09 | Black Magic | A82 | Sky line | A228 | Madamu Figaro |
| A10 | Carola | A83 | Golden Emblem | A232 | Margret Merril |
| A12 | Mirandy | A85 | Shinsei | A249 | Yntevose |
| A16 | Samantha | A86 | Marlorie Atherton | A252 | Jing Yu |
| A18 | Unknown1 | A88 | Princess Michael of Kent | A254 | Bolero |
| A21 | Shu-oh | A91 | Cocktail 80 | A255 | Dukat |
| A22 | Red cap | A92 | Golden Starlite | A256 | Athena |
| A23 | Red One | A93 | Goldmarie | A257 | Flair |
| A24 | Royal Class | A95 | Papillon | A258 | Vanilla Sky |
| A25 | Black Lady | A96 | Sonrisa | A260 | Moonlight Lover |
| A26 | Charles Mallerin | A97 | Flair | A261 | Lover Island |
| A27 | Cardinal Hume | A98 | Xiangzilan | A271 | Amsterdam |
| A28 | Precious Platinum | A101 | Sun Flare | A274 | Abaisite |
| A29 | Black Baccara | A102 | Golden Gate | A275 | Huang Ying |
| A41 | Diana | A104 | Violina | A276 | Green Tea |
| A42 | Naomi | A108 | Leandra | A278 | Sun City |
| A43 | Rhapsody | A109 | Azfran | A281 | Molineux |
| A44 | Vanity | A110 | Annemarie | A294 | Spanish Dancer |
| A45 | Yuzen | A111 | Iceberg | A307 | Nongkeyuan1 |
| A46 | Parade | A112 | Viviane | B125 | Amorosa |
| A47 | Fenshan | A115 | Avalanche | B302 | BJFU-27 |
| A51 | Universe | A116 | Ankila（White Universe） | B314 | Crimson Glory |
| A52 | Anna | A117 | Tineke | OK | Margo Koster |
| A53 | Lisa | A183 | Norita | PK | Pink Margo Koster |
| A54 | Belami | A186 | American Pride | RK | Red Margo Koster |
| A55 | Rosita Vendela | A187 | Barkarole | WK | White Margo Koster |
